# Cases and deaths from severe acute respiratory syndrome caused by COVID-19 in Indigenous populations: a descriptive study, Brazil, 2020-2021

**DOI:** 10.1590/S2237-96222025v34e20240630.en

**Published:** 2026-03-09

**Authors:** Yure Rodrigues Araújo Martins, Gerson Oliveira Penna, Fabíola Cristina Ribeiro Zucchi, Mauro Niskier Sanchez

**Affiliations:** 1 Universidade de Brasília, Faculdade de Medicina, Brasília, DF, Brazil; 2 Ministério da Saúde, Secretaria de Saúde Indígena, Brasília, DF, Brazil

**Keywords:** Health Services, Indigenous, Epidemiologic Surveillance Services, Severe Acute Respiratory Syndrome, COVID-19, Epidemiology Descriptive., Servicios de Salud del Indígena, Servicios de Vigilancia Epidemiológica, Síndrome Respiratorio Agudo Grave, COVID-19, Epidemiología Descriptiva.

## Abstract

**Objective:**

To assess the incidence, mortality and epidemiological-demographic profile of severe acute respiratory syndrome and deaths among confirmed COVID-19 cases in Indigenous populations between 2020 and 2021.

**Methods:**

This is a descriptive study based on COVID-19 data provided by the Ministry of Health’s Indigenous Health Secretariat. COVID-19 incidence and mortality in Brazil were analyzed. The analysis included creation of graphs and maps and calculation of hospitalization rates.

**Results:**

Incidence of severe cases varied between the Indigenous Health Districts (116.3 to 2,034.1 per 100,000 inhabitants), with the highest rates in the Kaiapó do Pará (2,034.1 per 100,000 inhabitants), Leste de Roraima (1,632.6 per 100,000 inhabitants) and Alto Rio Juruá (1,601.8 per 100,000 inhabitants) Districts. Comparing 2020 and 2021, there was a 64.9% reduction in the number of severe cases, from 4,520 in 2020 to 1,585 in 2021. In 2020, 27.1% of deaths occurred without hospitalization, decreasing to 18.8% in 2021. The majority of severe cases occurred in the 20-39 age group (30.6% in 2020 and 30.7% in 2021), and the highest concentration was in the Northern region of Brazil (69.7% in 2020 and 55.0% in 2021).

**Conclusion:**

The high incidence of severe cases and the high proportion of deaths without hospitalization highlight challenges in combating COVID-19 among Indigenous populations. The concentration of cases in the Northern region reinforces the need for specific strategies for this region.

## Resumen

**Objetivo:**

Evaluar la incidencia, la mortalidad y el perfil epidemiológico-demográfico del síndrome respiratorio agudo severo y las muertes en casos confirmados de COVID-19 en poblaciones indígenas entre 2020 y 2021.

**Métodos:**

Estudio descriptivo basado en datos de COVID-19 de la Secretaría de Salud Indígena del Ministerio de Salud. Se analizaron la incidencia y la mortalidad por COVID-19 en Brasil. El análisis incluyó la creación de gráficos y mapas, y el cálculo de las tasas de hospitalización.

**Resultados:**

La incidencia de casos graves varió entre los Distritos de Salud Indígenas (116,3 a 2.034,1 por 100.000 habitantes), con las tasas más altas en los Distritos de Kaiapó do Pará (2.034,1 por 100.000 habitantes), Este de Roraima (1.632,6 por 100.000 habitantes) y Alto Rio Juruá (1.601,8 por 100.000 habitantes). En comparación con 2020 y 2021, se observó una reducción del 64,9% en el número de casos graves, de 4.520 en 2020 a 1.585 en 2021. En 2020, el 27,1% de las muertes se produjeron sin hospitalización, cifra que disminuyó al 18,8% en 2021. La mayoría de los casos graves se presentaron en el grupo de edad de 20 a 39 años (30,6% en 2020 y 30,7% en 2021), y la mayor concentración se registró en la región Norte (69,7% en 2020 y 55,0% en 2021).

**Conclusión:**

La alta incidencia de casos graves y la alta proporción de muertes sin hospitalización ponen de relieve los desafíos en la lucha contra la COVID-19 entre las poblaciones indígenas. La concentración de casos en la región Norte refuerza la necesidad de estrategias específicas para esta región.

**Ethical aspects**

This research respected ethical principles, having obtained the following approval data:

Research ethics committee: National Research Ethics Committee

Opinion number: 6,627,394

Approval date: 09/2/2024

Certificate of submission for ethical appraisal: 71246822.9.0000.5558

Informed consent record: Not applicable

## Introduction

Occupation of Indigenous territories is a process of resistance, a struggle for cultural and territorial identity and the right to life. The relationship between the Indigenous population and their territories is marked by historical events of colonization, violence and land grabbing ^1^ ^,^ ^2^. The Indigenous population has been growing. In 2010, there were 817,963 Indigenous people in Brazil, with 315,000 living in urban areas and 502,963 in rural areas ^3^. In 2022, this number increased to 1,694,836, with 53.9% in urban areas and 46.1% in Indigenous territories ^4^. In Indigenous lands, the Indigenous health care system seeks to guarantee comprehensive and differentiated health care for Indigenous peoples ^5^ ^,^ ^6^.

The Ministry of Health’s Special Indigenous Health Secretariat is responsible for managing primary health care and water and sanitation. Special Indigenous Health Districts are administrative and operational units that provide health care services and implement water supply systems, respecting traditional knowledge and practices ^1^ ^,^ ^5^. Health information on Indigenous populations is recorded on the Indigenous Health Care Information System, which serves as a tool for health care and epidemiological surveillance actions ^1^ ^,^ ^5^_._

Despite the operational challenges of organizing services in geographically difficult-to-access locations, the information system monitors demographic, healthcare and epidemiological data on morbidity and mortality in contexts of both availability and lack of access to the internet, consolidating health indicators such as maternal and infant mortality rates and the percentage of pregnant women with access to prenatal care ^7^. However, with the COVID-19 Public Health Emergency of International Concern declared by the World Health Organization on February 3, 2020, and the urgent need for information, it became necessary to create a new epidemiological surveillance system to allow for timely variable changes and enable the reporting of COVID-19 cases in the Indigenous population ^7^ ^,^ ^8^.

In Brazil, the first case of COVID-19 was confirmed on February 26, 2020, before the official declaration of a public health emergency and the identification of community transmission ^9^. Among the territories served by the Indigenous health care system, the first confirmed case was recorded on March 13, 2020 ^10^ ^,^ ^11^. The health emergency exacerbated preexisting structural problems in Indigenous territories, aggravated by the absence of policies to prevent the exploitation of Indigenous lands and the dissemination of misinformation, such as fake news about the ineffectiveness of vaccines. These conditions contributed to vaccine refusal and an increase in invasions of Indigenous lands, illegal activities and deforestation ^12^ ^,^ ^10^, these being practices that increased the risk of SARS-CoV-2 infection among Indigenous populations ^13^ ^,^ ^14^.

Indigenous peoples faced challenges accessing health care during the COVID-19 emergency, exacerbated by socioeconomic, cultural and structural factors. Socioeconomic disparities and the recurring violation of territorial rights compounded logistical travel difficulties, precarious health facility infrastructure, high turnover of health professionals, and insufficient or nonexistent training for professionals working in intercultural contexts, which also negatively impacted Indigenous health services ^10^ ^,^ ^11^ ^,^ ^12^ ^,^ ^13^ ^e^ ^14^.

Considering the lack of comprehensive studies on COVID-19 among Indigenous populations using national data held by the Indigenous Health Secretariat, as well as the need to promote knowledge that contributes to the improvement of surveillance and health care strategies, with a view to greater equity in Indigenous health care, this study aims to evaluate the incidence, mortality and epidemiological-demographic profile of severe acute respiratory syndrome and deaths among confirmed cases of COVID-19 in Indigenous populations between 2020 and 2021.

## Methods

### Design

This was a descriptive observational study of incidence ^16^, to assess the magnitude and spatial distribution of severe acute respiratory syndrome (SARS) cases and deaths due to COVID-19 in Indigenous populations between 2020 and 2021.

### Setting

The 34 Special Indigenous Health Districts are strategic units of the Indigenous Health Care Subsystem, organized nationally based on geographic, ethnic and cultural criteria. They are distributed throughout the country and are present in every state, with 25 located in the Brazilian Legal Amazon and nine in regions outside the Amazon. They are structured into Base Hubs, subdivisions with territorial and operational functions, which coordinate and implement health actions on Indigenous lands. They serve approximately 800,000 Indigenous people in traditional territories. An estimated 54.0% of the Indigenous population (914,746 people) live in urban areas, while 46.0% live in non-urban areas and are under the direct responsibility of the Districts ^3^.

### Participants

The study population included Indigenous people living in traditional territories, classified as confirmed COVID-19 cases, with severe acute respiratory syndrome, or with a fatal outcome. Indigenous people living in urban areas were excluded, but cases of Indigenous people living in traditional territories treated at referral hospitals were included. Confirmed cases of COVID-19 were defined as individuals with laboratory confirmation of SARS-CoV-2 infection, through a positive RT-PCR or rapid antigen test, regardless of presence of symptoms ^16^.

Influenza-like illness is defined by the presence of at least two symptoms, such as fever (≥37.8°C), chills, sore throat, cough, headache, myalgia or arthralgia. Severe cases include these symptoms associated with signs of severity, such as oxygen saturation ≤95% or respiratory distress, with criteria adapted by age group according to the Ministry of Health’s Epidemiological Surveillance Guide. Deaths were confirmed based on COVID-19 as the underlying cause of death, using ICD-10 codes U07.1 and B34.2 ^16^.

### Variables

Demographic (age group; sex), geographic (region; health district), clinical (hospitalization; death), and temporal (epidemiological week of symptom onset; year of notification) variables were analyzed. Age groups (in years) were classified as 0-1, 1-4, 5-9, 10-14, 15-19, 20-39, 40-59, 60-79, and ≥80, and sex was classified as male and female. The geographic analysis included the region of Brazil (North; Northeast; Midwest; Southeast; South) and the 34 Indigenous Health Districts. Clinical variables analyzed hospitalization (presence or absence of hospitalization) and death as the outcome (confirmed death). Temporal variables included the epidemiological week of symptom onset and the year of notification (2020 or 2021).

### Data sources and measurement

The data were extracted from the COVID-19 platform created by the Indigenous Health Secretariat. Reported cases included records from the e-SUS system and the Influenza Surveillance System for the certification of severe cases and the provision of death certificates for confirmation of deaths, with validation also based on verification of laboratory results submitted by the Health Districts.

Population data were taken from the 2020 and 2021 database of the official system of the Indigenous Health Secretariat. Nominal population data comes from population registration performed by health teams in the territories and is constantly updated in primary health care processes.

### Statistical methods

The data were analyzed using the Statistical Package for the Social Sciences and Excel to organize the information and produce absolute and relative frequencies and rates. Quantum geographic information system software was also used to create maps. Tables of absolute and relative frequencies, bar graphs and incidence and mortality maps were used to present the distribution of demographic and epidemiological variables.

The incidence rates of severe acute respiratory syndrome due to COVID-19 and COVID-19 mortality per 100,000 inhabitants were calculated. The incidence rate was obtained by dividing the number of new cases per year by the population of each District during the same period, then multiplying the result by 100,000 inhabitants. The mortality rate was calculated by dividing the number of COVID-19 deaths during the period by the local population, also multiplied by 100,000 inhabitants.

The researchers checked the variables for completeness, and any inconsistencies were corrected. Duplicate cases were eliminated, and records were adjusted to keep clinical progression up to date.

## Results

In 2020 and 2021, a total of 6,105 cases of severe acute respiratory syndrome due to COVID-19 were recorded in Indigenous populations, as described in Table 1. In 2020, there were 4,520 cases, while in 2021, there were 1,585.

**Table 1:** Cases and percentages of severe acute respiratory syndrome due to COVID-19 among Indigenous village populations, according to variables. Brazil, 2020-2021

Variables	2020	2021	Total
	n (%)	n (%)	n (%)
Sex			
Female	2,356 (52.1)	817 (51.5)	3,173 (52.0)
Male	2,164 (47.9)	768 (48.5)	2,932 (48.0)
Age group (years)			
0-1	89 (2.0)	84 (5.3)	173 (2.8)
01/abr	181 (4.0)	133 (8.4)	314 (5.1)
05/set	101 (2.2)	55 (3.5)	156 (2.5)
out/14	119 (2.6)	55 (3.4)	174 (2.9)
15-19	209 (4.6)	94 (5.9)	303 (5.0)
20-39	1,385 (30.7)	488 (30.8)	1,873 (30.7)
40-59	1,305 (28.9)	355 (22.4)	1,660 (27.2)
60-79	813 (18.0)	216 (13.7)	1.029 (16.9)
80+	318 (7.0)	105 (6.6)	423 (6.9)
Region			
Midwest	458 (10.1)	181 (11.4)	639 (10.5)
Northeast	644 (14.2)	261 (16.5)	905 (14.8)
North	3,149 (69.7)	871 (55.0)	4,020 (65.8)
Southeast	52 (1.2)	53 (3.3)	105 (1.7)
South	217 (4.8)	219 (13.8)	436 (7.1)
Hospitalization			
No	3,888 (86.0)	1,249 (78.8)	5,136 (84.1)
Yes	632 (14.0)	336 (21.2)	968 (15.9)
Total	4,520 (74.0)	1,585 (26.0)	6,105 (100.0)

Regarding hospitalizations, we found that in 2020, part of the cases (14.0%) had access to hospitalization. This percentage increased in 2021 (15.9%). However, one finding of this study is the high proportion of deaths that occurred without access to specialized care services. In 2020, 27.1% of deaths (more than a quarter) due to severe acute respiratory syndrome were not preceded by hospitalization. Although deaths without hospitalization reduced to 18.8% in 2021, the cumulative total for 2020-2021 reveals that 24.1% of deaths among Indigenous people occurred without hospital admission.

Most severe cases occurred in individuals between 20 and 39 years of age (30.6% in 2020; 30.7% in 2021). In 2020 and 2021, the majority of SARS cases due to COVID-19 occurred in Indigenous women, accounting for 52.1% of cases in 2020 and 51.5% in 2021 (total of 52.0%). Despite this, deaths were more frequent among men, who accounted for 65.3% of deaths in 2020 and 53.8% in 2021 (total of 61.2%). Geographically, the Northern region concentrated the majority of cases (69.7% in 2020; 55.0% in 2021), followed by the Northeast region. Most cases did not require or did not have access to hospitalization (86.0% in 2020; 78.8% in 2021) (Table 2).

**Table 2:** Deaths and percentages of severe acute respiratory syndrome due to COVID-19 among Indigenous village populations, according to variables. Brazil, 2020-2021

Variables	2020	2021	Total
	n (%)	n (%)	n (%)
Sex			
Female	201 (34.7)	148 (46.3)	349 (38.8)
Male	378 (65.3)	172 (53.7)	550 (61.2)
Age group (years)			
0-1	17 (2.9)	11 (3.4)	28 (3.1)
01/abr	4 (0.7)	10 (3.1)	14 (1.6)
05/set		4 (1.3)	4 (0.4)
out/14	1 (0.2)	1 (0.3)	2 (0.2)
15-19	7 (1.2)	3 (0.9)	10 (1.1)
20-39	32 (5.6)	32 (10.0)	64 (7.1)
40-59	110 (19.0)	66 (20.6)	176 (19.6)
60-79	226 (39.0)	109 (34.1)	335 (37.3)
80+	182 (31.4)	84 (26.3)	266 (29.6)
Region			
Midwest	182 (31.4)	57 (17.8)	239 (26.6)
Northeast	67 (11.6)	60 (18.7)	127 (14.1)
North	271 (46.8)	141 (44.1)	412 (45.8)
Southeast	9 (1.6)	13 (4.1)	22 (2.4)
South	50 (8.6)	49 (15.3)	99 (11.0)
Hospitalization			
No	157 (27.1)	60 (18.8)	217 (24.1)
Yes	422 (72.9)	260 (81.2)	682 (75.9)
Total	579 (64.4)	320 (35.6)	899 (100.0)

**Figure 1:**
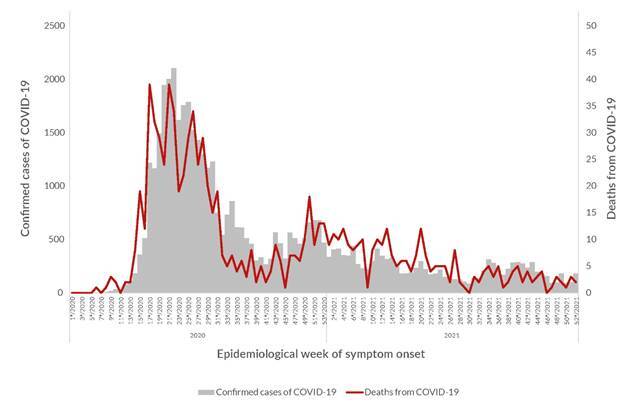
Number of confirmed COVID-19 cases and deaths among Indigenous village populations by epidemiological week of symptom onset. Brazil, 2020-2021

Figure 1 shows the distribution of confirmed COVID-19 cases and deaths over the epidemiological weeks of 2020 and 2021. A temporal analysis of COVID-19 severe acute respiratory syndrome cases (Figure 1) demonstrates a peak in severe cases during epidemiological weeks 18 to 32 of 2020. The data demonstrate a temporal correlation between the increase in confirmed cases and deaths, with peaks occurring in periods similar to those observed in the previous figure. A second peak in cases can be seen in late 2020 and early 2021, followed by periods of increasing cases of influenza-like illness without an increase in severe cases.

**Figure 2.**
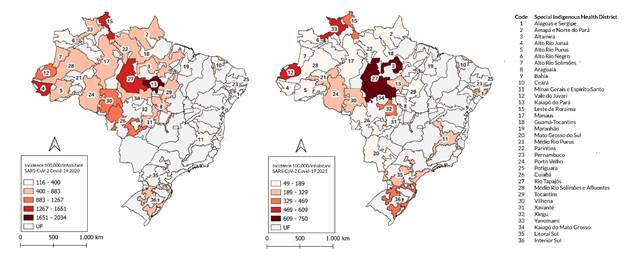
Incidence rates per 100,000 inhabitants of severe acute respiratory syndrome due to COVID-19 among Indigenous village populations, according to Special Indigenous Health District. Brazil, 2020-2021

The incidence map (Figure 2) demonstrates the occurrence of confirmed severe acute respiratory syndrome due to COVID-19 distributed by Health District in 2020 and 2021. The data reveal variations between Health Districts, with some recording much higher rates. In 2020, the Alto Rio Juruá District in Acre had 1,601.8 cases per 100,000 inhabitants, followed by the Leste de Roraima District, with 1,632.6 cases/100,000 inhabitants, and the Kaiapó District of Pará, with the highest rate, 2,034.1 cases/100,000 inhabitants. In contrast, the Health Districts of Mato Grosso do Sul, Minas Gerais, and Espírito Santo recorded much lower rates, namely 116.3 and 182.6 cases per 100,000 inhabitants, as shown in Table 3.

**Table 3:** Number of cases and incidence rates of severe acute respiratory syndrome and COVID-19 mortality rates per 100,000 inhabitants among Indigenous village populations, according to Special Indigenous Health District. Brazil, 2020-2021

Special Indigenous Health District	2020	2021	2020	2021
	Severe acute respiratory syndrome-COVID-19			COVID-19 mortality	
	n (rate)	n (rate)	n (rate)	n (rate)
Alagoas and Sergipe	22 (170.1)	33 (255.1)	5 (38.7)	5 (38.6)
Altamira	41 (875.1)	36 (749.7)	2 (42.7)	
Alto Rio Juruá	295 (1,601.8)	26 (136.0)	10 (54.3)	1 (5.2)
Alto Rio Negro	100 (342.8)	19 (68.6)	17 (58.3)	16 (57.8)
Alto Rio Purus	83 (639.2)	9 (78.1)	5 (38.5)	3 (26.0)
Alto Rio Solimões	441 (602.0)	64 (89.9)	37 (50.5)	16 (22.5)
Amapá and Norte do Pará	66 (497.3)	16 (115.9)	4 (30.1)	3 (21.7)
Araguaia	45 (769.0)	4 (69.8)	7 (119.6)	
Bahia	105 (303.0)	60 (172.2)	10 (28.9)	7 (20.1)
Ceará	123 (442.6)	86 (324.2)	11 (39.6)	19 (71.6)
Cuiabá	94 (1,219.2)	15 (186.9)	24 (311.3)	6 (74.8)
Guamá Tocantins	137 (710.7)	14 (59.8)	17 (88.2)	4 (17.1)
Interior Sul	176 (439.8)	147 (390.4)	42 (105.0)	39 (103.6)
Kaiapó do Mato Grosso	34 (659.8)	42 (655.4)	5 (97.0)	
Kaiapó do Pará	124 (2,034.1)	12 (190.0)	10 (164.0)	1 (15.8)
Leste de Roraima	891 (1,632.6)	227 (403.5)	61 (111.8)	49 (87.1)
Litoral Sul	61 (232.7)	78 (312.4)	15 (57.2)	13 (52.1)
Manaus	151 (461.6)	58 (183.3)	17 (52.0)	9 (28.4)
Maranhão	140 (331.0)	20 (48.6)	27 (63.8)	14 (34.0)
Mato Grosso do Sul	93 (116.3)	47 (58.7)	79 (98.8)	33 (41.2)
Médio Rio Purus	25 (303.0)	21 (326.3)	5 (60.6)	2 (22.5)
Médio Rio Solimões and Tributaries	94 (416.3)	18 (87.0)	12 (53.1)	2 (9.7)
Minas Gerais and Espírito Santo	32 (182.6)	47 (265.8)	2 (11.4)	10 (56.5)
Parintins	84 (495.5)	14 (84.4)	12 (70.8)	7 (42.2)
Pernambuco	69 (169.1)	38 (89.4)	10 (24.5)	11 (25.9)
Porto Velho	67 (609.9)	23 (203.4)	8 (72.8)	6 (53.1)
Potiguara	175 (1,098.6)	24 (141.0)	4 (25.1)	4 (23.5)
Rio Tapajós	189 (1,366.3)	94 (651.1)	17 (122.9)	8 (55.4)
Tocantins	90 (697.7)	16 (121.9)	10 (77.5)	1 (7.6)
Vale do Javari	64 (974.1)	36 (568.2)	2 (30.4)	1 (15.8)
Vilhena	74 (1,228.0)	13 (204.4)	15 (248.9)	5 (78.6)
Xavante	134 (584.7)	35 (147.0)	49 (213.8)	10 (42.0)
Xingu	31 (376.5)	34 (400.0)	15 (182.2)	5 (58.8)
Yanomami	170 (592.0)	159 (537.2)	13 (45.3)	10 (33.8)
Grand total	4,520 (580.1)	1,585 (202.9)	579 (74.3)	320 (41.0)

In 2021, most Districts saw a decline in COVID-19 incidence rates. The Alto Juruá River District saw a significant reduction to 136.0 cases per 100,000 inhabitants, and the rate in the Lest de Roraima District, although still high, fell to 403.5 cases per 100,000 inhabitants. The Kaiapó District of Pará also saw a reduction to 190.0 cases per 100,000 inhabitants. In Altamira, the reduction was smaller, from 875.1 to 749.7 cases per 100,000 inhabitants. Severe cases continued to predominate in the Rio Tapajós District (651.1 cases per 100,000), the Kaiapó District of Mato Grosso (655.4 cases per 100,000), the Vale do Javari District (568.2 cases per 100,000), and the Yanomami District (537.2 cases per 100,000).

**Figure 3:**
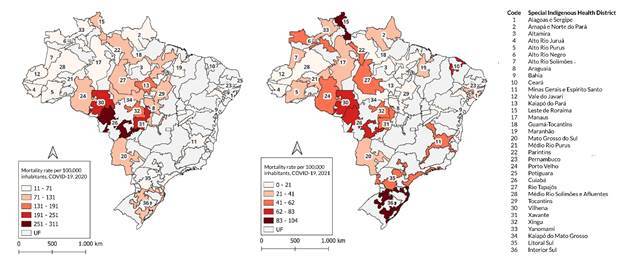
COVID-19 mortality rates per 100,000 inhabitants among Indigenous village populations, according to Special Indigenous Health District. Brazil, 2020-2021

Figure 3 highlights variations in COVID-19 mortality between Districts, with distinct distributions of severe cases. In 2020, mortality rates were concentrated in the Tapajós River (122.9/100,000), Vilhena (248.9/100,000), Kaiapó do Pará (164.0/100,000), and Xavante (213.8/100,000) Health Districts, which accounted for the highest number of severe cases. However, in 2021, the Districts with the highest number of severe cases also had high mortality rates, particularly in the Alto Rio Negro (57.8/100,000), Interior Sul (103.6/100,000), and Xingu (58.8/100,000) Districts.

## Discussion

Between 2020 and 2021, during the COVID-19 public health emergency, a low proportion of severe hospitalized cases was observed among Indigenous populations, and a significant reduction in the total number of severe cases in Indigenous territories was observed. However, the proportion of severe cases among adults aged 20 to 39 increased during this period, which may be related to several factors, such as greater occupational exposure, greater exposure to urban environments, as well as lower adherence to preventive measures. Similarly, an increase in the percentage of severe cases among males was observed, while a decrease was seen among females, possibly influenced by behavioral differences.

At the same time, there was a reduction in the proportion of severe cases that did not have access to or did not require access to hospitals. Regarding mortality, there was a reduction in deaths among men from 2020 to 2021, with the absolute number of deaths among adults aged 20 to 39 remaining stable overall, and particularly in the Southern region of Brazil. The highest concentration of severe cases among Indigenous populations was recorded in the Legal Amazon region.

This reduction in the total number of severe cases may be associated with prevention and control measures implemented in Indigenous territories during this period, such as inclusion of the Indigenous population in the priority group for immunization, expansion of rapid antigen test distribution, provision of rapid response health teams, and the contact tracing strategy ^16^. However, the increased proportion of severe cases among young adults may indicate differences in exposure to the virus, possibly related to this age group’s greater involvement in subsistence activities or external contact. The reduction in severe cases without access to hospitals suggests greater coordination between Indigenous health teams and municipal and state referral services. However, the concentration of cases in the Legal Amazon reinforces the need to prioritize actions in this region, given its historical vulnerability and geographic barriers to accessing health services ^10^.

The study has limitations, such as the small number of RT-PCR tests in Indigenous territories, compromising diagnostic accuracy ^16^. Indigenous people living in urban areas and cases of influenza-like or severe respiratory syndromes not suspected as being COVID-19 or without access to rapid tests or examinations were excluded from the reported cases. Deaths without confirmation of COVID-19 as the underlying cause were also discarded. Furthermore, surveillance, developed during the emergency, faced underreporting due to complex regional flows and uneven infrastructure, impacting data completeness and accuracy ^10^.

The intersection between the epidemiological scenario in Indigenous territories and COVID-19 incidence may have increased the risk of respiratory complications. In areas with difficult geographic access, limited availability of intensive care units may restrict the capacity to treat severe COVID-19 cases. This scenario highlights the need for strategies to train health professionals, promote health communication, vaccinate, continuously detect cases of influenza-like illnesses, identify at-risk patients, triage them, and ensure timely transfer to referral hospitals ^17^ ^,^ ^18^ ^e^ ^19^ ^).^

Despite the proportion of non-hospitalized patients, most deaths among Indigenous people occurred in hospitalized patients, with the Northern region recording the highest concentration of these outcomes. Mortality rates were higher in 2020, but in 2021, redistribution of deaths was observed, particularly in Health Districts such as Alto Rio Negro and Interior Sul. This pattern coincides with the initial period of high transmissibility and extensive spread of COVID-19, prior to the implementation of vaccination campaigns operated by the Ministry of Health ^9^ ^e^ ^10^.

The incidence and mortality data reveal significant variations between Health Districts. In 2020, territories such as Alto Rio Juruá and Kaiapó do Pará recorded the highest rates. In 2021, a general downward trend was observed in these indicators. Even so, some Districts maintained high rates of severe cases and mortality. Descriptive exploration identified areas of higher risk, highlighting priority regions for public health actions. These patterns may be related to the circulation of SARS-CoV-2 variants, with greater transmissibility and lower case fatality ratios ^17^.

Among the factors that can interfere with the response to public health emergencies, considering the COVID-19 epidemiological scenario, we highlight the need for active surveillance and compliance with protocols, with widespread testing of Indigenous populations and healthcare professionals. Such measures enable expanded detection capacity, meaning healthcare facilities can identify cases among symptomatic and asymptomatic individuals, facilitating rapid isolation of cases and their contacts ^12^ ^e^ ^13^.

Adopting appropriate preventive care is essential to prevent health services from acting as potential amplifiers of virus transmission ^18^ ^e^ ^19^. In vulnerable conditions, especially in border areas or where there are isolated and recently contacted Indigenous populations, preventive measures and protocols become even more essential to mitigate the spread of COVID-19 and reduce the incidence of severe cases with death as their outcome. In this context, this study emphasizes the importance of soft technologies, given the susceptibility of Indigenous populations to outbreaks of respiratory diseases, and reinforces the importance of effective control and prevention strategies ^9^ ^e^ ^16^.

High transmission of the virus in 2020 and 2021, combined with insufficient implementation of control measures in Brazil, posed a persistent threat to Indigenous communities. In the Legal Amazon, collection of epidemiological data and identification of severe cases faced structural and logistical challenges due to delays in information processing. The proportional reduction in cases in the Northern region in 2021 may reflect both changes in transmission and gaps in reporting ^19^.

The limited infrastructure of the Base Hubs, which are operational healthcare units in Indigenous territories, in addition to the vast distances traveled by health teams and the lack of logistical resources, such as aircraft, vessels, fuel, and sufficient flight hours, compromises the presence of teams in the territories and adequate collection of samples for analysis in the state-level Central Public Health Laboratories, hindering accurate diagnoses and genomic identification of the virus. Furthermore, use of rapid tests and issuance of clear guidelines for health professionals and Indigenous communities face the challenge of respecting the intercultural context, given the great ethnocultural, regional, environmental and social diversity of these populations, which impedes the standardization of strategies at the national level ^10^ ^,^ ^14^.

The COVID-19 spread patterns in Indigenous populations expose health inequalities, weaknesses in surveillance, overlapping emergencies, precarious socio-environmental conditions, and limited preventive actions and campaign operational models. The increase in mortality during periods of high transmissibility highlights these challenges, requiring further studies ^12^ ^e^ ^13^. The persistence of COVID-19 in Indigenous territories poses an additional threat, adding to the high disease burden these populations already face, such as acute diarrheal diseases, malaria and other infectious and parasitic diseases ^20^. Furthermore, prevalence of comorbidities, such as chronic diseases and malnutrition, further increases the risk of severe forms of COVID-19 in Indigenous populations ^21^.

Incorporating COVID-19 incidence into the epidemiological profile of Indigenous territories demonstrated increased risk of respiratory complications. This challenge is reflected in the study data, which indicate a distinct distribution of COVID-19 cases and mortality across regions, with the Northern region recording the highest concentration of severe cases and possibly being the region most prone to under-services due to the absence or inequitable distribution of higher-educated health professionals, which is exacerbated by limited infrastructure, long shifts in the area, and the long distances health teams have to travel ^22^ ^,^ ^23^ ^e^ ^24^.

The high mortality rates observed in certain Districts reinforce the need to expand access and promote adaptations in intensive care offered by referral units to accommodate Indigenous populations, in addition to strengthening the healthcare infrastructure of Indigenous Health Centers. It is equally essential to improve information flows between Indigenous health units and integrate data systems at the national level. Active communication between Health District managers and healthcare networks and inter-managerial committees in strategic municipalities is also necessary to consolidate processes and ensure more agile and effective care for Indigenous populations. These gaps reinforce the urgent need to prepare Indigenous Health Districts for future public health emergencies related to respiratory viruses, in a manner integrated with state and municipal Health Departments, thus reducing mortality and ensuring coordinated and more assertive responses ^10^ ^,^ ^13^ ^,^ ^19^ ^,^ ^23^ ^e^ ^24^.

Despite the overall reduction in incidence and mortality, possibly a reflection of control measures and vaccination, some regions maintained high rates even a year after the end of the public health emergency. Therefore, continuous monitoring of vaccination coverage and the strengthening of adapted prevention and control strategies are essential, with an emphasis on areas with difficult geographic access ^17^ ^e^ ^19^.

The results indicate a high number of deaths without hospitalization and an uneven distribution of severe cases and mortality among Indigenous Health Districts. Further studies are needed to understand the factors contributing to these deaths and severe cases. The implications for Indigenous health policies include the need to unveil fake news, the possibility of hospitalization, improvements in Indigenous health infrastructure, access to supplies and medicines, expanded testing and diagnosis, training and qualification of epidemiological surveillance practices, and analysis of Indigenous territory health data.
