# Supplementary material for: Cases and deaths from severe acute respiratory syndrome caused by COVID-19 in Indigenous populations: a descriptive study, Brazil, 2020-2021
Source: Epidemiol Serv Saude. 2026 Mar 9;35:e20240630. doi: 10.1590/S2237-96222025v34e20240630.en (PMC12970838; doi:10.1590/S2237-96222025v34e20240630.en)
Supplement: Translation

# Translation: Casos e óbitos da síndrome respiratória aguda grave causada por covid-19 nas populações indígenas: estudo descritivo, Brasil, 2020-2021

**DOI:** 10.1590/S2237-96222025v34e20240630.pt

**Authors:** Yure Rodrigues Araújo Martins, Gerson Oliveira Penna, Fabíola Cristina Ribeiro Zucchi, Mauro Niskier Sanchez

**Affiliations:** 1 Universidade de Brasília, Faculdade de Medicina, Brasília, DF, Brasil; 2 Ministério da Saúde, Secretaria de Saúde Indígena, Brasília, DF, Brasil

**Keywords:** Serviços de Saúde do Indígena, Serviços de Vigilância Epidemiológica, Síndrome Respiratória Aguda Grave, Covid-19, Epidemiologia Descritiva.

## Abstract

**Objetivo:**

Avaliar a incidência, a mortalidade e o perfil epidemiológico-demográfico da síndrome respiratória aguda grave e dos óbitos entre casos confirmados de covid-19 de populações indígenas entre 2020 e 2021.

**Métodos:**

Estudo descritivo baseado em dados de covid-19 da Secretaria de Saúde Indígena do Ministério da Saúde. Foram analisadas incidência e mortalidade por covid-19 no Brasil. A análise contemplou a elaboração de gráficos e mapas e o cálculo das proporções de hospitalização.

**Resultados:**

A incidência de casos graves variou entre os Distritos Sanitários Indígenas (116,3-2.034,1/100.000 habitantes), com as maiores taxas nos distritos Kaiapó do Pará (2.034,1/100.000 habitantes), Leste de Roraima (1.632,6/ 100.000 habitantes) e Alto Rio Juruá (1.601,8/ 100.000 habitantes). No comparativo entre 2020 e 2021, observou-se redução de 64,9% no número de casos graves, passando de 4.520 em 2020 para 1.585 em 2021. Em 2020, 27,1% dos óbitos ocorreram sem hospitalização, reduzindo para 18,8% em 2021. A maioria dos casos graves ocorreu na faixa etária de 20-39 anos (30,6% em 2020 e 30,7% em 2021), e a maior concentração foi na região Norte (69,7% em 2020 e 55,0% em 2021).

**Conclusão:**

A alta incidência de casos graves e a elevada proporção de óbitos sem hospitalização evidenciam desafios no enfrentamento da covid-19 entre populações indígenas. A concentração de casos na região Norte reforça a necessidade de estratégias específicas para essa região.

**Aspectos éticos**

Esta pesquisa respeitou os princípios éticos, obtendo os seguintes dados de aprovação:

Comitê de ética em pesquisa: Comissão Nacional de Ética em Pesquisa

Número do parecer: 6.627.394

Data de aprovação: 09/2/2024

Certificado de apresentação de apreciação ética: 71246822.9.0000.5558

Registro do consentimento livre e esclarecido: Não se aplica

## Introdução

A ocupação dos territórios indígenas é um processo de resistência, de luta pela identidade cultural, territorial e pelo direito à vida. A relação entre a população indígena e seus territórios é marcada por eventos históricos de colonização, violência e usurpação de terras ^1^ ^e^ ^2^. A população indígena tem crescido. Em 2010, havia 817.963 indígenas no Brasil, com 315 mil vivendo em áreas urbanas e 502.963 em áreas rurais ^3^. Em 2022, esse número aumentou para 1.694.836, com 53,9% em áreas urbanas e 46,1% em territórios indígenas ^4^. Nas terras indígenas, o sistema de atenção à saúde indígena busca garantir a atenção integral e diferenciada à saúde dos povos indígenas ^5^ ^e^ ^6^.

A Secretaria Especial de Saúde Indígena do Ministério da Saúde é responsável pela gestão da atenção primária à saúde e pelo abastecimento de água e saneamento. Os Distritos Sanitários Especiais Indígenas são unidades administrativas e operacionais que executam serviços de atenção à saúde e a implantação de sistemas de abastecimento de água, respeitando saberes e práticas tradicionais ^1^ ^e^ ^5^. As informações de saúde das populações indígenas são registradas no Sistema de Informações da Atenção à Saúde Indígena, que é ferramenta para as ações de assistência e de vigilância epidemiológica ^1^ ^e^ ^5^.

Apesar dos desafios operacionais para a organização dos serviços em localidades de difícil acesso geográfico, o sistema de informações monitora dados demográficos, assistenciais e epidemiológicos de morbidade e mortalidade em contexto de ausência e de disponibilidade de internet, consolidando indicadores de saúde, como taxa de mortalidade materno-infantil e percentual de gestantes com acesso ao pré-natal ^7^. No entanto, com a Emergência de Saúde Pública de Importância Internacional por covid-19, declarada pela Organização Mundial da Saúde em 3 de fevereiro de 2020, e a necessidade urgente de informações, tornou-se necessária a criação de um novo sistema de vigilância epidemiológica para permitir a alteração de variáveis em tempo oportuno, a fim de viabilizar a notificação de casos de covid-19 na população indígena ^7^ ^e^ ^8^.

No Brasil, o primeiro caso de covid-19 foi confirmado em 26 de fevereiro de 2020, antes da declaração oficial de emergência de saúde pública e da identificação de transmissão comunitária ^9^. Entre os territórios atendidos pelo sistema de atenção à saúde indígena, o primeiro caso confirmado foi registrado em 13 de março de 2020 ^10^ ^e^ ^11^. A emergência sanitária exacerbou problemas estruturais preexistentes nos territórios indígenas, agravados pela ausência de políticas para impedir a exploração de terras indígenas e pela disseminação de desinformações, como notícias falsas sobre a ineficácia das vacinas. Essas condições contribuíram para a recusa de vacinas e para o aumento de invasões nas terras indígenas, de atividades ilegais e de desmatamento ^12^ ^e^ ^10^, práticas que elevaram o risco de infecção por SARS-CoV-2 entre as populações indígenas ^13^ ^e^ ^14^.

Os povos indígenas enfrentaram desafios no acesso à atenção à saúde durante a emergência por covid-19, agravados por fatores socioeconômicos, culturais e estruturais. Disparidades socioeconômicas e a recorrente violação de direitos territoriais somaram-se às dificuldades logísticas de deslocamento, à precariedade de infraestrutura de unidades de saúde, à alta rotatividade de profissionais de saúde e à insuficiente ou inexistente capacitação dos profissionais para atuação em contextos interculturais, o que também impactou negativamente os serviços de saúde indígena ^10^ ^,^ ^11^ ^,^ ^12^ ^,^ ^13^ ^e^ ^14^.

Considerando a ausência de estudos abrangentes sobre a covid-19 entre populações indígenas utilizando dados nacionais da Secretaria de Saúde Indígena, bem como a necessidade de promover conhecimentos que contribuam para o aprimoramento das estratégias de vigilância e de assistência, com vistas a uma maior equidade na atenção à saúde indígena, este estudo propõe-se a avaliar a incidência, a mortalidade e o perfil epidemiológico-demográfico da síndrome respiratória aguda grave e dos óbitos entre casos confirmados de covid-19 em populações indígenas entre 2020 e 2021.

## Métodos 

### Desenho do estudo

Este foi um estudo descritivo observacional de incidência ^16^, para avaliar a magnitude e a distribuição espacial de casos de síndrome respiratória aguda grave e de óbitos por covid-19 nas populações indígenas entre 2020 e 2021.

### Contexto

Os 34 Distritos Sanitários Especiais Indígenas são unidades estratégicas do Subsistema de Atenção à Saúde Indígena, organizadas nacionalmente com base em critérios geográficos, étnicos e culturais. Eles estão distribuídos por todas as regiões do país e presentes em todos estados, com 25 localizados na Amazônia Legal e nove em regiões extra-amazônicas. Estruturam-se em Polos Base, subdivisões com função territorial e operacional, que coordenam e executam ações de saúde nas terras indígenas. Atendem cerca de 800 mil indígenas em territórios tradicionais. Estima-se que 54,0% da população indígena (914.746 pessoas) viva em áreas urbanas, enquanto 46,0% sejam residentes em áreas não urbanas, e estejam sob responsabilidade direta dos distritos ^3^.

### Participantes

A população do estudo incluiu indígenas residentes em territórios tradicionais, classificados como casos confirmados de covid-19, com síndrome respiratória aguda grave ou com desfecho de óbito. Foram excluídos os indígenas residentes em áreas urbanas, mas incluídos casos de indígenas que vivem nos territórios tradicionais atendidos em unidades hospitalares de referência. Casos confirmados de covid-19 foram definidos como indivíduos com confirmação laboratorial de infecção por SARS-CoV-2, através de RT-PCR positivo ou de teste rápido com detecção de antígenos, independentemente da presença de sintomas ^16^.

A síndrome gripal é definida pela presença de pelo menos dois sintomas, como febre (≥37,8°C), calafrios, dor de garganta, tosse, dor de cabeça, mialgia ou artralgia. Casos graves incluem esses sintomas associados a sinais de gravidade, como saturação ≤95% ou dificuldade respiratória, com critérios adaptados por faixa etária conforme o *Guia de Vigilância Epidemiológica* do Ministério da Saúde. Os óbitos foram confirmados com base na causa básica de morte por covid-19, utilizando os códigos da décima Classificação Internacional de Doenças (CID): U07.1 e B34.2 ^16^.

### Variáveis

Foram analisadas variáveis demográficas (faixa etária; sexo), geográficas (região; distrito), clínicas (hospitalização; evolução para óbito) e temporais (semana epidemiológica de início dos sintomas; ano de notificação). As faixas etárias (em anos) foram classificadas como 0-1, 1-4, 5-9, 10-14, 15-19, 20-39, 40-59, 60-79 e ≥80, e o sexo foi classificado em masculino e feminino. A análise geográfica envolveu a região (Norte; Nordeste; Centro-Oeste; Sudeste; Sul) e os 34 distritos. As variáveis clínicas analisaram a hospitalização (presença ou ausência de internação) e a evolução para óbito (óbito confirmado). As variáveis temporais englobaram a semana epidemiológica de início dos sintomas e o ano de notificação (2020 ou 2021).

### Fonte de dados e mensuração

Os dados foram extraídos da plataforma covid-19, criada pela Secretaria de Saúde Indígena. Notificações incluíram registros do e-SUS e do Sistema de Vigilância da Gripe para homologação de casos graves e a disponibilização de declarações de óbito para confirmação dos óbitos, com validação baseada também na verificação dos resultados laboratoriais enviados pelos distritos. 

Os dados populacionais foram utilizados a partir da base de dados de 2020 e 2021 do sistema oficial da Secretaria de Saúde Indígena. Os dados populacionais nominais são advindos do cadastramento das equipes de saúde nos territórios e atualizados constantemente nos processos de atenção primária à saúde.

### Métodos estatísticos

Os dados foram analisados utilizando o software Statistical Package for the Social Sciences e Excel para organizar as informações e produzir as frequências absolutas e relativas e as taxas. Além disso, utilizou-se o software do sistema de informações geográficas Quantum para elaboração de mapas. Tabelas de frequências absolutas e relativas, gráficos de barras e mapas de incidência e de mortalidade foram utilizados para apresentar a distribuição das variáveis demográficas e epidemiológicas. 

Foram calculadas as taxas de incidência de síndrome respiratória aguda grave por covid-19 e de mortalidade por covid-19 por 100 mil habitantes. A taxa de incidência foi obtida dividindo-se o número de casos novos por ano pela população de cada distrito no mesmo período, multiplicando-se o resultado por 100 mil habitantes. Já a taxa de mortalidade foi calculada pela divisão entre o número de óbitos por covid-19 no período e a população do local, também multiplicada por 100 mil habitantes.

A completude das variáveis foi verificada pelos pesquisadores, e as inconsistências foram corrigidas. Casos duplicados foram eliminados, e os registros foram ajustados, a fim de manter a evolução clínica atualizada. 

## Resultados

Ao longo de 2020 e 2021, foram registrados 6.105 casos de síndrome respiratória aguda grave por covid-19 em populações indígenas (Tabela 1). Em 2020, contabilizaram-se 4.520 casos, enquanto, em 2021, o número foi de 1.585 casos.

**Tabela 1:** Casos e percentual de síndrome respiratória aguda grave por covid-19 entre populações indígenas aldeadas por variáveis. Brasil, 2020-2021

Variáveis	2020	2021	Total
	n (%)	n (%)	n (%)
Sexo			
Feminino	2.356 (52,1)	817 (51,5)	3.173 (52,0)
Masculino	2.164 (47,9)	768 (48,5)	2.932 (48,0)
Faixa etária (anos)			
0-1	89 (2,0)	84 (5,3)	173 (2,8)
01/abr	181 (4,0)	133 (8,4)	314 (5,1)
05/set	101 (2,2)	55 (3,5)	156 (2,5)
out/14	119 (2,6)	55 (3,4)	174 (2,9)
15-19	209 (4,6)	94 (5,9)	303 (5,0)
20-39	1.385 (30,7)	488 (30,8)	1.873 (30,7)
40-59	1.305 (28,9)	355 (22,4)	1.660 (27,2)
60-79	813 (18,0)	216 (13,7)	1.029 (16,9)
80+	318 (7,0)	105 (6,6)	423 (6,9)
Região			
Centro-Oeste	458 (10,1)	181 (11,4)	639 (10,5)
Nordeste	644 (14,2)	261 (16,5)	905 (14,8)
Norte	3.149 (69,7)	871 (55,0)	4.020 (65,8)
Sudeste	52 (1,2)	53 (3,3)	105 (1,7)
Sul	217 (4,8)	219 (13,8)	436 (7,1)
Hospitalização			
Não	3.888 (86,0)	1.249 (78,8)	5.136 (84,1)
Sim	632 (14,0)	336 (21,2)	968 (15,9)
Total	4.520 (74,0)	1.585 (26,0)	6.105 (100,0)

No que concerne às hospitalizações, observou-se que, em 2020, uma parcela dos casos (14,0%) teve acesso à internação hospitalar. Esse percentual apresentou aumento em 2021 (15,9%). Contudo, um achado deste estudo foi a proporção elevada de óbitos que ocorreram sem acesso a serviços de atenção especializada. Em 2020, 27,1% dos óbitos por síndrome respiratória aguda grave não foram precedidos por hospitalização, ou seja, mais de um quarto dos óbitos. Embora tenha havido uma redução para 18,8% dos óbitos em 2021, o total acumulado para o biênio 2020-2021 revelou que 24,1% dos óbitos em indígenas ocorreram sem que houvesse internação hospitalar.

A maioria dos casos graves ocorreu em indivíduos entre 20 anos e 39 anos (30,6% em 2020; 30,7% em 2021). Em 2020 e 2021, a maioria dos casos de SRAG por covid-19 ocorreu em indígenas do sexo feminino, com 52,1% dos casos em 2020 e 51,5% em 2021 (total de 52,0%). Apesar disso, os óbitos foram mais frequentes entre homens, que representaram 65,3% das mortes em 2020 e 53,8% em 2021 (total de 61,2%). Geograficamente, a região Norte concentrou a maior parte dos casos (69,7% em 2020; 55,0% em 2021), sendo seguida pela região Nordeste. A maioria dos casos não necessitou ou não teve acesso à hospitalização (86,0% em 2020; 78,8% em 2021) (Tabela 2). 

**Tabela 2:** Óbitos e percentual por síndrome respiratória aguda grave por covid-19 entre populações indígenas aldeadas por variáveis. Brasil, 2020-2021

Variáveis	2020	2021	Total
	n (%)	n (%)	n (%)
Sexo			
Feminino	201 (34,7)	148 (46,3)	349 (38,8)
Masculino	378 (65,3)	172 (53,7)	550 (61,2)
Faixa etária (anos)			
0-1	17 (2,9)	11 (3,4)	28 (3,1)
01/abr	4 (0,7)	10 (3,1)	14 (1,6)
05/set		4 (1,3)	4 (0,4)
out/14	1 (0,2)	1 (0,3)	2 (0,2)
15-19	7 (1,2)	3 (0,9)	10 (1,1)
20-39	32 (5,6)	32 (10,0)	64 (7,1)
40-59	110 (19,0)	66 (20,6)	176 (19,6)
60-79	226 (39,0)	109 (34,1)	335 (37,3)
80+	182 (31,4)	84 (26,3)	266 (29,6)
Região			
Centro-Oeste	182 (31,4)	57 (17,8)	239 (26,6)
Nordeste	67 (11,6)	60 (18,7)	127 (14,1)
Norte	271 (46,8)	141 (44,1)	412 (45,8)
Sudeste	9 (1,6)	13 (4,1)	22 (2,4)
Sul	50 (8,6)	49 (15,3)	99 (11,0)
Hospitalização			
Não	157 (27,1)	60 (18,8)	217 (24,1)
Sim	422 (72,9)	260 (81,2)	682 (75,9)
Total	579 (64,4)	320 (35,6)	899 (100,0)

Observou-se a distribuição dos casos confirmados de covid-19 e dos óbitos ao longo das semanas epidemiológicas de 2020 e 2021 (Figura 1). A análise temporal dos casos de síndrome respiratória aguda grave por covid-19 (Figura 1) demonstrou um pico de casos graves durante as semanas epidemiológicas 18 a 32 de 2020. Os dados demonstraram uma correlação temporal entre o aumento dos casos confirmados e os óbitos, com picos ocorrendo em períodos semelhantes aos observados na figura anterior. Um segundo pico de casos foi observado no final de 2020 e início de 2021, seguidos de períodos de aumento de casos de síndromes gripais sem o aumento de casos graves.

**Figura 1:** Quantidade de casos confirmados e de óbitos por covid-19 entre populações indígenas aldeadas por semana epidemiológica de início dos sintomas. Brasil, 2020-2021

O mapa (Figura 2) de incidência demonstra a ocorrência de síndrome respiratória aguda grave confirmada por covid-19 distribuída por distrito em 2020 e 2021. Os dados revelam variações entre os distritos sanitários, com alguns registrando taxas muito mais altas. Em 2020, o distrito Alto Rio Juruá, no Acre, teve 1.601,8 casos por 100 mil habitantes, seguido pelo distrito Leste de Roraima, com 1.632,6 casos/100 mil habitantes e o distrito Kaiapó do Pará, com a maior taxa, 2.034,1 casos/100 mil habitantes. Em contrapartida, os distritos de Mato Grosso do Sul, Minas Gerais e Espírito Santo registraram taxas bem mais baixas, de 116,3 e de 182,6 casos por 100 mil habitantes, conforme Tabela 3.

**Figura 2:** Taxas de incidência por 100 mil habitantes de síndrome respiratória aguda grave por covid-19 entre populações indígenas aldeadas por Distrito Sanitário Especial Indígena. Brasil, 2020-2021

**Tabela 3:** Número de casos, taxas de incidência de síndrome respiratória aguda grave e taxas de mortalidade por 100 mil habitantes de covid-19 entre populações indígenas aldeadas por Distrito Sanitário Especial Indígena. Brasil, 2020-2021

Distrito Sanitário Especial Indígena	2020	2021	2020	2021
	Incidência de síndrome respiratória aguda grave/covid-19			Mortalidade por covid-19	
	n (taxa)	n (taxa)	n (taxa)	n (taxa)
Alagoas e Sergipe	22 (170,1)	33 (255,1)	5 (38,7)	5 (38,6)
Altamira	41 (875,1)	36 (749,7)	2 (42,7)	
Alto Rio Juruá	295 (1.601,8)	26 (136,0)	10 (54,3)	1 (5,2)
Alto Rio Negro	100 (342,8)	19 (68,6)	17 (58,3)	16 (57,8)
Alto Rio Purus	83 (639,2)	9 (78,1)	5 (38,5)	3 (26,0)
Alto Rio Solimões	441 (602,0)	64 (89,9)	37 (50,5)	16 (22,5)
Amapá e Norte do Pará	66 (497,3)	16 (115,9)	4 (30,1)	3 (21,7)
Araguaia	45 (769,0)	4 (69,8)	7 (119,6)	
Bahia	105 (303,0)	60 (172,2)	10 (28,9)	7 (20,1)
Ceará	123 (442,6)	86 (324,2)	11 (39,6)	19 (71,6)
Cuiabá	94 (1.219,2)	15 (186,9)	24 (311,3)	6 (74,8)
Guamá Tocantins	137 (710,7)	14 (59,8)	17 (88,2)	4 (17,1)
Interior Sul	176 (439,8)	147 (390,4)	42 (105,0)	39 (103,6)
Kaiapó do Mato Grosso	34 (659,8)	42 (655,4)	5 (97,0)	
Kaiapó do Pará	124 (2.034,1)	12 (190,0)	10 (164,0)	1 (15,8)
Leste de Roraima	891 (1.632,6)	227 (403,5)	61 (111,8)	49 (87,1)
Litoral Sul	61 (232,7)	78 (312,4)	15 (57,2)	13 (52,1)
Manaus	151 (461,6)	58 (183,3)	17 (52,0)	9 (28,4)
Maranhão	140 (331,0)	20 (48,6)	27 (63,8)	14 (34,0)
Mato Grosso do Sul	93 (116,3)	47 (58,7)	79 (98,8)	33 (41,2)
Médio Rio Purus	25 (303,0)	21 (326,3)	5 (60,6)	2 (22,5)
Médio Rio Solimões e Afluentes	94 (416,3)	18 (87,0)	12 (53,1)	2 (9,7)
Minas Gerais e Espírito Santo	32 (182,6)	47 (265,8)	2 (11,4)	10 (56,5)
Parintins	84 (495,5)	14 (84,4)	12 (70,8)	7 (42,2)
Pernambuco	69 (169,1)	38 (89,4)	10 (24,5)	11 (25,9)
Porto Velho	67 (609,9)	23 (203,4)	8 (72,8)	6 (53,1)
Potiguara	175 (1.098,6)	24 (141,0)	4 (25,1)	4 (23,5)
Rio Tapajós	189 (1.366,3)	94 (651,1)	17 (122,9)	8 (55,4)
Tocantins	90 (697,7)	16 (121,9)	10 (77,5)	1 (7,6)
Vale do Javari	64 (974,1)	36 (568,2)	2 (30,4)	1 (15,8)
Vilhena	74 (1.228,0)	13 (204,4)	15 (248,9)	5 (78,6)
Xavante	134 (584,7)	35 (147,0)	49 (213,8)	10 (42,0)
Xingu	31 (376,5)	34 (400,0)	15 (182,2)	5 (58,8)
Yanomami	170 (592,0)	159 (537,2)	13 (45,3)	10 (33,8)
Total	4.520 (580,1)	1.585 (202,9)	579 (74,3)	320 (41,0)

Em 2021, a maioria dos distritos apresentou queda nas taxas de incidência de covid-19. O Alto Rio Juruá reduziu expressivamente para 136,0 casos/100 mil habitantes, e o Leste de Roraima, embora ainda alto, caiu para 403,5 casos/100 mil habitantes. O Kaiapó do Pará também reduziu para 190,0 casos/100 mil habitantes. Em Altamira, a redução foi menor, de 875,1 para 749,7 casos/100 mil habitantes. Casos graves continuaram predominando nos distritos Rio Tapajós (651,1/100 mil), Kaiapó do Mato Grosso (655,4/100 mil), Vale do Javari (568,2/100 mil) e Yanomami (537,2/100 mil).

A Figura 3 destaca variações na mortalidade por covid-19 entre distritos, com distribuição distinta da incidência de casos graves. Em 2020, é possível observar que a concentração da mortalidade no Rio Tapajós (122,9/100 mil), Vilhena (248,9/100 mil) Kaiapó do Pará (164,0/100 mil) e Xavante (213,8/100 mil) correspondem à ocorrência dos casos graves. No entanto, no ano de 2021, os distritos com maior número de casos graves também concentraram alta mortalidade, destacando-se os distritos Alto Rio Negro (57,8/100 mil), Interior Sul (103,6/100 mil) e Xingu (58,8/100 mil).

**Figura 3:** Taxas de mortalidade por 100 mil habitantes de covid-19 entre populações indígenas aldeadas por Distrito Sanitário Especial Indígena. Brasil, 2020-2021

## Discussão

Entre 2020 e 2021, durante a emergência de saúde pública por covid-19, foi evidenciada uma baixa proporção de casos graves hospitalizados entre as populações indígenas e observada uma grande redução no número total de casos graves nos territórios indígenas. Contudo, a proporção de casos graves entre adultos de 20 anos a 39 anos apresentou um aumento no período, o que pode estar relacionado a diversos fatores, como maior exposição ocupacional, maior exposição ao contexto urbano e menor adesão às medidas preventivas. Da mesma forma, observou-se um crescimento no percentual de casos graves no sexo masculino, enquanto no feminino houve uma redução, possivelmente influenciada por diferenças comportamentais. 

Paralelamente, registrou-se uma redução na proporção de casos graves que não tiveram acesso ou não precisaram ter acesso às unidades hospitalares. Em relação à mortalidade, houve redução nos óbitos entre homens no período de 2020 a 2021, com manutenção do número absoluto de óbitos entre adultos de 20 anos a 39 anos no contexto geral e, principalmente, na região Sul. A maior concentração de casos graves entre as populações indígenas foi registrada na região da Amazônia Legal.

Essa redução no número total de casos graves pode estar associada a medidas de prevenção e de controle implementadas nos territórios indígenas durante o período, como a inclusão da população indígena no grupo prioritário para imunização, a ampliação da distribuição de testes rápidos de antígeno, a disponibilização de equipes de saúde de resposta rápida e a estratégia de rastreamento de contatos ^16^. No entanto, o aumento da proporção de casos graves entre adultos jovens pode indicar diferenças de exposição ao vírus, possivelmente relacionadas ao maior envolvimento dessa faixa etária em atividades de subsistência ou de contato externo. A redução de casos graves sem acesso hospitalar sugere maior articulação entre as equipes de saúde indígena e os serviços municipais e estaduais de referência. No entanto, a concentração de casos na Amazônia Legal reforça a necessidade de priorizar ações nessa região, dada a sua vulnerabilidade histórica e as barreiras geográficas para o acesso a serviços de saúde ^10^.

O estudo apresenta limitações, como o reduzido número de exames RT-PCR nos territórios indígenas, comprometendo a precisão diagnóstica ^16^. As notificações excluem indígenas em áreas urbanas e casos de síndromes gripais ou respiratórias graves não suspeitas de covid-19 ou sem acesso a testes rápidos ou exames. Óbitos sem confirmação básica de covid-19 também foram desconsiderados. Além disso, a vigilância, desenvolvida durante a emergência, enfrentou subnotificação devido a fluxos regionais complexos e infraestrutura desigual, impactando a completude e a precisão dos dados ^10^.

A interseção do cenário epidemiológico nos territórios indígenas com a incidência de covid-19 pode ter aumentado o risco de complicações respiratórias. Em áreas de difícil acesso geográfico, a disponibilidade limitada de unidades de terapia intensiva pode restringir a capacidade de atendimento a casos graves de covid-19. Esse cenário destaca a necessidade de estratégias de capacitação de profissionais de saúde, de comunicação em saúde, de vacinação, de detecção contínua de casos de síndromes gripais, de identificação de pacientes em risco, de triagem e de transferência oportuna para unidades hospitalares de referência ^17^ ^,^ ^18^ ^e^ ^19^.

Apesar da proporção de não hospitalizados, a maioria dos óbitos entre indígenas ocorreu em pacientes hospitalizados, com a região Norte registrando a maior concentração desses desfechos. As taxas de mortalidade foram mais elevadas em 2020, mas, em 2021, observou-se uma redistribuição dos óbitos, com destaque para regiões como Alto Rio Negro e Interior Sul. Esse padrão coincide com o período inicial de alta transmissibilidade e de ampla disseminação da covid-19, anterior à implementação das ações de vacinação operacionalizadas pelo Ministério da Saúde ^9^ ^e^ ^10^.

Os dados de incidência e de mortalidade evidenciam variações importantes entre os distritos. Em 2020, territórios como Alto Rio Juruá e Kaiapó do Pará registraram as maiores taxas. Em 2021, observou-se uma tendência geral de redução desses indicadores. Ainda assim, alguns distritos mantiveram taxas elevadas de casos graves e de mortalidade. A exploração descritiva identificou áreas de maior risco, apontando regiões prioritárias para ações em saúde pública. Esses padrões podem estar relacionados à circulação de variantes do SARS-CoV-2, com maior transmissibilidade e menor letalidade ^17^.

Entre os fatores que podem interferir na resposta às emergências de saúde pública, considerando o cenário epidemiológico da covid-19, destacam-se a necessidade de vigilância ativa e o cumprimento de protocolos, com ampla realização de testes nas populações indígenas e nos profissionais de saúde. Tais medidas possibilitam a ampliação da capacidade de detecção, ou seja, a possibilidade de as unidades de saúde promoverem a identificação de casos entre indivíduos sintomáticos e assintomáticos, facilitando o isolamento rápido de casos e de seus contatos ^12^ ^e^ ^13^.

A adoção de cuidados preventivos adequados é fundamental para evitar que os serviços de saúde atuem como potenciais amplificadores da transmissão do vírus ^18^ ^e^ ^19^. Em condições de vulnerabilidade, especialmente em áreas de fronteira ou com a presença de populações indígenas isoladas e de recente contato, medidas preventivas e protocolares tornam-se ainda mais essenciais para mitigar a disseminação da covid-19 e reduzir a incidência de casos graves de óbitos. Nesse contexto, este estudo enfatiza a importância de tecnologias leves, diante da suscetibilidade das populações indígenas a surtos de doenças respiratórias, e reforça a relevância de estratégias eficazes de controle e de prevenção ^9^ ^e^ ^16^.

A elevada transmissão do vírus em 2020 e 2021, aliada à implementação insuficiente de medidas de controle no Brasil, representou uma ameaça persistente às comunidades indígenas. Na Amazônia Legal, a coleta de dados epidemiológicos e a identificação de casos graves enfrentaram desafios estruturais e logísticos pelo atraso no processamento de informações. A redução proporcional de casos na região Norte em 2021 pode refletir tanto mudanças na transmissão quanto lacunas na notificação ^19^.

A infraestrutura limitada dos Polos Base, que são unidades operacionais de atenção à saúde nos territórios indígenas, além das vastas distâncias percorridas pelas equipes de saúde e da carência de recursos logísticos, como aeronaves, embarcações, combustível e horas de voo suficientes, comprometem a presença das equipes nos territórios e a coleta adequada de amostras para análise nos Laboratórios Centrais de Saúde Pública, dificultando diagnósticos precisos e a identificação genômica do vírus. Além disso, a utilização de testes rápidos e a emissão de orientações claras para profissionais de saúde e comunidades indígenas enfrentam o desafio de respeitar o contexto intercultural, dada à grande diversidade étnico-cultural, regional, ambiental e social dessas populações, o que impede a padronização de estratégias em âmbito nacional ^10^ ^e^ ^14^.

Os padrões de dispersão da covid-19 em populações indígenas expõem desigualdades na saúde, fragilidades na vigilância, sobreposição de emergências, condições socioambientais precárias e ações preventivas limitadas e em modelos operacionais de campanhas. O aumento da mortalidade em períodos de alta transmissibilidade ressalta esses desafios, exigindo novos estudos ^12^ ^e^ ^13^. A persistência da covid-19 nos territórios indígenas representa uma ameaça adicional, somando-se à alta carga de doenças que essas populações já enfrentam, como doenças diarreicas agudas, malária e outras enfermidades infecciosas e parasitárias ^20^. Além disso, a prevalência de comorbidades, como doenças crônicas e desnutrição, elevaram ainda mais o risco de formas graves de covid-19 nas populações indígenas ^21^.

A incorporação da incidência de covid-19 no perfil epidemiológico dos territórios indígenas aumentou o risco de complicações respiratórias. Esse desafio é refletido nos dados do estudo, que apontam para uma distribuição distinta dos casos e da mortalidade por covid-19 entre as regiões, com a região Norte registrando a maior concentração de casos graves, sendo possivelmente a região mais sujeita a desassistência pela ausência ou pela falta de distribuição equitativa de profissionais de saúde de nível superior, o que é exacerbado pela infraestrutura limitada, pelas longas escalas de permanência em área e pelas grandes distâncias a serem percorridas pelas equipes de saúde ^22^ ^,^ ^23^ ^e^ ^24^.

A elevada mortalidade observada em determinados distritos reforça a necessidade de ampliar o acesso e de promover adaptações nos cuidados intensivos ofertados pelas unidades de referência, a fim de contemplar as populações indígenas, além de fortalecer a infraestrutura assistencial das Casas de Saúde Indígena. É igualmente essencial aprimorar os fluxos de informação entre as unidades de saúde indígena e integrar os sistemas de dados em nível nacional. A comunicação ativa dos gestores distritais com as redes de atenção à saúde e com as comissões intergestores em municípios estratégicos também é procedimento necessário para consolidar processos e garantir um atendimento mais ágil e eficaz às populações indígenas. Essas lacunas reforçam a urgência de preparar os distritos para futuras emergências de saúde pública relacionadas a vírus respiratórios, de forma integrada aos estados e municípios, reduzindo, assim, a mortalidade e assegurando respostas coordenadas e mais assertivas ^10^ ^,^ ^13^ ^,^ ^19^ ^,^ ^23^ ^e^ ^24^.

Apesar da redução geral na incidência e na mortalidade, possivelmente reflexo das medidas de controle e da vacinação, algumas regiões mantiveram altas taxas, mesmo após um ano do término da emergência de saúde pública. Diante disso, torna-se essencial o monitoramento contínuo das coberturas vacinais e o fortalecimento de estratégias adaptadas de prevenção e de controle, com ênfase nas áreas de difícil acesso geográfico ^17^ ^,^ ^19^.

Os resultados indicam alto número de óbitos sem hospitalização e distribuição desigual de casos graves e de mortalidade entre distritos sanitários indígenas. Novos estudos são necessários para entender os fatores que contribuíram para os óbitos e os casos graves. As implicações para as políticas de saúde indígena incluem a necessidade de esclarecimento de notícias falsas, a possibilidade de hospitalização, a melhoria nas infraestruturas de saúde indígena, o acesso a insumos e medicamentos, a ampliação da testagem e do diagnóstico, o treinamento e a qualificação das práticas de vigilância epidemiológica e a análise de dados de saúde dos territórios indígenas.
